# Letter to Dr. E. S. Frazer, from His Son, S. H. Frazer, in Paris

**Published:** 1867-12

**Authors:** 


*Letter to* DR. E. S. FRAZER, *from his son*, S. H. FRAZER, *in Paris.—The General Pathology of Syphilis—Letter III. —The Indurated Chancre.*

It is not necessary to repeat here what was said in the two former letters concerning the indurated chancre. We have seen that it is the first local manifestation of the disease proper, and, therefore, only a symptom. I deem it proper to say, in this connection, that the first writers on syphilis did not confound the two chancres; for we find that Marcellus Cumanus and Alexander Benedictus made a distinction between the indurated chancre and the contagious ulcer of the genital organs. I have tried to show that the first writers who confounded the two were Georgis Vella and Nicholas Massa. Other syphilographers, however, were struck with the difference existing in appearance and prognosis; they noticed these differences without attaching any importance to them. Jean de Vigo speaks of the induration ordinarily accompanying the chancre, followed by constitutional syphilis. Thierry de Hèry, in 1569, wrote that "these ulcers are the most often accompanied by hardness; the greater the degree of hardness, the greater the degree of malignity and difficulty of cure; the prognosis will also be more doubtful." Ambroise Parè wrote the following, about the same epoch: "If there is an ulcer on the penis, and induration occurs, it invariably proves that the patient has syphilis." Hunter noticed, and gave a most classic description concerning this induration. M. Ricord was the first who seized it clearly, but he taught that all chancres can become indurated if placed in certain conditions. M. Bassereau, of Paris, enjoys the honor of having divided the two, and made up the differential characters existing between them.

*Cause.*—I have already said, in the former letters, that constitutional syphilis always commences with a chancre; also, that this chancre can arise from different sources. M. Rollet teaches and writes that all secondary symptoms of the suppurating form are contagious, making the pus the vehicle of the virus. As already seen, the blood of a syphilitic patient is also contagious; but syphilographers have not as yet been able to determine in what condition.

*Symptomatology.*—The majority of writers have agreed upon twenty-five days as the average of the incubation; it was formerly taught that it made its debut by a vesicle, but at present, it is taught that it makes its appearance by a simple excoriation, the diameter of which is not larger than the head of a pin of ordinary size. It is round, covered (except on its borders) by a grayish pseudo-membranous production, of which the presence is constant. It suppurates very little, progressively augments in surface, and passes successively through the stage known as that of

"*state*" and reparation. The false membrane remains generally on a level with the neighboring tissues; it neither forms a prominence nor depression. The borders of the ulcer are of a bright red; not covered by the false membrane, they (the edges) look as if varnished—shining. That peculiar arrangement of the borders, towards the bottom of the ulcer, gives it that cup-shaped form, in view of which M. Ricord has so much insisted they do not resemble the borders of the soft chancre. However, there are some cases where the borders are raised, appearing to be perpendicularly excavated; then it has almost the same appearance as the soft chancre.

The induration of the base of a chancre generally makes its appearance during the first week following the apparition of the ulcer; sometimes it is much longer making its appearance. Fifteen days, and even three weeks, elapse before the induration can be detected; it is almost always difficult to ascertain, at the commencement, whether it is an induration. It develops slowly and progressively until up to the moment of cicatrization of the chancre; then it gradually diminishes, after a time, which is very variable; it (the cicatrization) can be found six months after the disappearance of the chancre. Ricord says, in his Lessons on the Chancre, (page 106,) that he found the induration of a cicatrix thirty years after the healing of the chancre. When it has reached its maximum, it forms a sort of bed for the ulcer; it is indurated around and under the ulcer. The induration which belongs to a large ulceration is often badly marked; this gives (from its feel) origin to M. Ricord's pasteboard induration. In some cases, only the borders are indurated; it is then called circular; sometimes it acquires a considerable volume, forms a hard, cartilaginous-like body, which raises the ulcer above the neighboring tissues; this the authors name *ulcus elevatum.* After the teachings of MM. Diday and Langlebert, the typical chancre is the result of contagion from a primary accident, and the *pasteboard* chancre the result of contagion from a secondary accident. These views have a great many friends among syphilographers. The induration, no matter what form it takes, seems to have certain seats of election, and produces itself more easily in those regions. Thus, on man we see the preputial chancres; those on the *corona glandis*, in the cervix or gutter between the prepuce and glans, indurate ordinarily very well; the cutaneous chancres on the body of the penis indurate badly. Among women, the induration is very obscure; the induration, when once established, has a tendency to disappear, whether the patient be submitted to treatment or not; it softens with this peculiarity, namely, throughout its extent and thickness, disappearing little by little, and finally leaves no trace behind but a violet-colored spot, which will in time disappear. A very frequent complication is the ulceration of the induration, which, in some cases, takes place, and would induce inexperienced persons to believe that they had a new chancre to deal with. It is probably this, says M. Bellehomme, that made Mr. Babington write that the primary accident of syphilis is constituted by an induration preceding the ulceration.

The following is a statistical table of M. Puche, giving the length of time the induration existed:

| | | | |
|---|---|---|---|
| Chancre | in Cervix | 390 | days. |
| .. | of Frenum | 452 | .. |
| .. | .. Cervix | 457 | .. |
| .. | .. Cervix | 540 | .. |
| .. | .. Cervix | 602 | .. |
| .. | .. Glans | 650 | .. |
| .. | .. Cervix | 690 | .. |
| .. | .. Prepuce | 700 | .. |
| .. | .. Prepuce | 755 | .. |
| .. | .. Cervix | 768 | .. |
| .. | .. Cervix | 997 | .. |
| .. | .. Cervix | 1507 | .. |
| .. | .. Prepuce | 2062 | .. |

I said that among women the induration is less marked than among men. It happens sometimes that there is no induration at all; such chancres are observed in the proportion to the others of one to one hundred. The diagnosis of this variety is difficult, but it is only necessary to remember that polyganglious induration always accompanies the indurated chancre and never the soft. If need be, inoculation can be tried on the person where the case is obscure; the ulcer will indicate the nature of the sore, whether chancre or chancroid.

The mixed chancre has made such a noise in the medical world, in the last few years, that a few remarks, I think, in this connection, will be appropriate. The theory was first promulgated by M. Laroyenne, a pupil of M. Rollet, published in 1859. M. Laroyenne having put some pus from a soft chancre on an indurated one, thus engrafting the soft on the hard chancre, the reunion was called by him "mixed chancre." The following year, M. Basset repeated the experiments of M. Laroyenne, and obtained the same results. The simultaneous cöexistence of the two kinds of virus on the same point explains, beyond a doubt, the cases in which pretended indurated chancres have been inoculated on the person who is afflicted with it; but this reinoculation has only been possible, according to the statistics of MM. Puche and Fournier, two cases in one hundred, and after M. Rollet, six in one hundred. The mixed chancre gives also a plausible interpretation of the observations of pretended soft chancres transmitting soft chancres, followed by constitutional symptoms. It is also an excellent argument in favor of duality, since this chancre furnishes an inoculable pus for the individual who is affected; this is, say dualists, a certain proof that the viruses are on the same place, and yet distinct, each preserving its specious properties. In general, the indurated chancre is solitary; according to statistics of M. Clerc, it is so in four or five cases; nor is this surprising when we remember that the indurated chancre does not, like the soft one, multiply by inoculation. It is necessary, in order to have more than one hard chancre, that more than one be contracted at the same time. M. Aimè Martin says: If an embarrassing case presents itself to the practitioner with a chancre, and eight or ten others arise afterwards, it should be diagnosticated as soft chancre. In the largest majority of cases, (ninety-three in one hundred,) the indurated chancre gives rise to engorgement of the lymphatic ganglions in the neighboring groin. These engorgements, which are called *polyganglia*, are multiple, hard, indolent, without any change of the color of the skin. The indurated ganglions are independent of each other, without adherence to the cellular tissue; they roll freely under the finger, not the least tendency to suppuration. It is very rare that the indurated chancre is complicated with *phagedenism* or gangreen, which, on the contrary, happens often with soft chancres. Acute inflammation seldom accompanies it, (the hard chancre;) in a word, the complications, in all their phases, are very rare. After having rested for a few days in the condition known as the "*state*," its tendency is towards cicatrization, which ordinarily is accomplished at the end of a month. When a chancre takes the *ulcus elevatum* form, it has, in appearance, the greatest analogy to mucous patches; they are very easily confounded. It can even happen that secondary accidents come to light during the process of cicatrization, and the chancre be transformed into a mucous patch. This transformation M. Ricord used in support of his theory of the non-contagiosity of secondary accidents. Even when it had been demonstrated that the pus from a mucous patch had transmitted syphilis, the Midi School never failed to use this argument; they said that the mucous patch which furnished the pus was nothing but a chancre under the process of transformation. The chancre has not any exclusive place of election; it flourishes at any point where the virus may be introduced. The genital organs being more exposed than other parts, of course are more frequently affected than others. MM. Bellehomme and Martin divide the chancre into two classes, according to the cause, the genital-indurated and extra-genital.

The genital-indurated chancre exists on an average of ninety-five in one hundred. M. Clerc's statistical table of four hundred and three cases of indurated chancres is as follows:

| | |
|---|---|
| Mucous surface of the prepuce | 63 |
| Cervix of furrow between glans and prepuce | 171 |
| Orifice of the prepuce | 35 |
| Frenum | 14 |
| Glans | 12 |
| Meatus urinarius | 33 |
| On the body of the penis | 58 |
| Scrotum | 3 |
| Angle formed by the penis and scrotum | 5 |

Of four hundred and seventy one cases of indurated chancres observed by M. Fournier, at the Midi, he has noted only twenty-six extra-genital chancres:

| | |
|---|---|
| Chancres of the glans and prepuce | 314 |
| Of the body of the penis | 60 |
| Multiples, 62; chancres at the same time of the body and prepuce, body and glans | 11 |

| | |
|---|---|
| Meatus urinarius | 32 |
| Inside of the urethra | 17 |
| Scrotum | 7 |
| Between the penis and scrotum | 4 |

In a statistical report of M. Rollet, at Lyons, extending from the 1st of November, 1862, to the 5th of March, 1863, we find, that of fifty-three indurated chancres, fifty were genital chancres, three presenting the character of mixed chancres. M. Nodet, an interne in the service of M. Rollet, observed, during six months, sixty-five indurated chancres, fifty-nine of which were on the genital organs.

M. Tanturrè, of the Syphilicime in Naples, has given the following in forty cases:

| | |
|---|---|
| Chancres of the glans | 4 |
| Cervix of penis | 6 |
| Mucous membrane of the prepuce | 16 |
| Orifice of the prepuce | 3 |
| Body of the penis | 7 |
| Scrotum | 2 |
| Pubis | 2 |

There are few statistics of the position of chancres among women; this is probably owing to the rapidity with which they disappear, and the malformation of the induration. M. Martin, an interne in the service of M. Clerc, has given a statistical table for the year 1861; of all the indurated chancres admitted in the St. Lazare Hospital, unfortunately, says M. Martin, these statistics can only have relatively a secondary value; for the majority of the patients who compose that service are prostitutes, having been formerly affected with syphilis, and, consequently, are exempt from a new contraction of an indurated chancre. Of the seven hundred and seventy-six patients affected with divers venereal affections, admitted from the 1st of January, 1861, to the 31st of December of the same year, forty-five were affected with indurated chancres, thirty-three genital, twelve extra-genital. The first he divides as follows:

| | |
|---|---|
| Chancres of the Labia majora | 15 |
| .. .. Labia minora | 9 |
| .. .. Fourchette | 5 |
| .. .. Meatus urinarius | 2 |
| .. .. Vestibule | 2 |

There was not a single case during this year of a chancre of the walls of the vagina or cervix uteri.

M. Melchior Robert has arrived at different results. Of seventy-six patients affected with chancre—he does not say whether they were indurated or not—on the external genital organs, he found:

| | |
|---|---|
| Fourchette | 30 |
| Limits of the vulva and vagina | 11 |
| Vulva | 7 |
| Labia minora | 17 |
| Meatus | 4 |
| Internal face of the labia majora | 3 |
| Clitoris | 2 |
| Caruncul[illegible]s | 2 |

Before passing to the statistics of the extra-genital chancres, I will say here a few words, following Bellehomme and Martin, of the concealed urethral chancre. The possibility of such a thing was noticed by Hernandez; afterwards it was specially studied by M. Ricord. In M. Fournier's statistics of four hundred and forty-five indurated chancres, he found seventeen which could only be perceived by forcibly separating the lips of the meatus. A patient affected at the same time with one of these and gonorrhœa would give at the same time syphilis and gonorrhœa. Persons contenting themselves with a superficial examination would only see the most apparent lesion, and, consequently, would see a beautiful example of syphilitic gonorrhœa. This order of things has been the refuge and harbor of the Identist.

The extra-genital chancre has only been noticed ten times in four hundred and three cases by M. Clerc, twenty-six in forty-five by Aimè Martin. The last, of M. Martin, was from the St. Lazare, and the disproportion is explained by the depraved habits of the prostitutes; as M. Tardieu says: "*Labia et oscula obscœnis blanditiis praebent.*"

The ten cases of M. Clerc are divided as follows:

| | |
|---|---|
| Lips | 5 |
| Tongue | 1 |
| Pubis | 2 |
| Thigh | 1 |
| Eye lid | 1 |

Of the twenty-six of M. Fournier:

| | |
|---|---|
| Anus | 6 |
| Lips | 12 |
| Tongue | 3 |
| Nose | 1 |
| Mucous membrane of the nose | 1 |
| Eye lid | 1 |
| Finger | 1 |
| Leg | 1 |

Four of M. Burtel:

| | |
|---|---|
| Lower lip | 2 |
| Both lips at once | 1 |
| Cephalic, without any other designation | 1 |

Of the six cases observed by M. Nodet:

| | | |
|---|---|---|
| Chancre of | Mucous membrane cervix | 1 |
| .. | Internal angle of right eye | 1 |
| .. | Lower lip | 4 |

The twelve cases observed at the St. Lazare:

| | |
|---|---|
| Perineum | 2 |
| Anus | 2 |
| Thigh | 1 |
| Buttock | 1 |
| Lower lip | 2 |
| Wing of the nose | 1 |
| Tongue | 1 |
| Base of uvula | 1 |
| Forehead | 1 |

The indurated chancre of the anus often escapes the attention of the practitioner; it is ordinarily a light and painless lesion; it does not often attract the attention of the patient. The buccal chancres, chancres of the tongue, lips and uvula, as comprised under this head, are most often the result of contagion from secondary accidents of the mouth; it can result very often in *mediate contagion.* M. Rollet reports a circumstance of transmission from mouth to mouth through the agency of a blowing tube in a glass factory in the department of the Loire. Chancres of the nose and nares are very rare. M. Fournier reports one of each, and Mr. McCarthy one of the nares. Those of the face occur more frequently. M. Bellehomme has seen one on the forehead, and another situated in the gutter between the nose and upper lip; M. Fournier has seen a similar one; M. Melchior Robert speaks of a chancre of the forehead; M. Ricord speaks of a medical student as having one on the left cheek; others have cited examples of chancres of the eyelid, among other, Ricord, Melchior Robert and Desmarres. It is very easy to be seen, from the above statistics, that the indurated chancre of the head occurs frequently enough, and this relative frequency, perhaps, accounts for the extreme rarity with which the soft chancre is met with in this region; indeed, the head was considered for a long time refractory to the virus of the soft chancre.

*Relative Frequency of the Indurated Chancre.*—M. Fournier, in his notes on Ricord's Lessons on the Chancre, says that he observed, at the Midi Hospital, during nine months, three hundred and forty-one chancres, of which

| | | |
|---|---|---|
| Indurated chancres were | 126 | 341 |
| Soft chancres were | 215 | |

M. Barlet, at the Antiquaille:

| | | |
|---|---|---|
| Indurated chancres | 54 | 131 |
| Soft chancres | 77 | |

M. Nodet:

| | | |
|---|---|---|
| Indurated chancres | 65 | 136 |
| Soft chancres | 71 | |

M. Bellehomme, at St. Lazare:

| | | |
|---|---|---|
| Indurated chancres | 45 | 150 |
| Soft chancres | 105 | |

The general condition of the system during the duration of the indurated chancre was an extremely remarkable phenomenon, and which goes to form the assertion that the chancre is only the first external manifestation of the diathesis. It is rare that the patient does not complain of an unaccustomed feebleness, of palpitation, headache, or show a discolorization of the integuments, and sometimes a "*bruit de souffle*" in the carotids; in a word, all the symptoms of chloro-anemia. Something more remarkable still, is the state of the blood, which has been studied and analyzed by M. Grossi, formerly pharmaceutist at the Hotel Dieu. This experimenter found, in all the cases, a considerable diminution of the red globules. According to MM. Becquerel and Rodier, the normal number of the red globules should be one hundred and forty in one thousand parts. In the several analyses of M. Grossi, the normal number of 140 was lessened to 125, 124, 95, 94, 90, 76, 58, 55, and even 48. On the other hand, the number of parts of albumen was increased (represented commonly by 80) to 102, 104, 106, 108, 115, 123, 126 and 127. The quantity of fibrin was not notably changed in any case. The blood of individuals affected with soft chancre was also examined by M. Grossi, but did not present any important alteration.

*Histological Nature of the Induration.*—M. Ricord thinks it is formed by an effusion of plastic lymph in the lymphatic capillary system. According to the theories of MM. Marchal de Colroi, Robin, Lebert and Mr. Acton, the induration belongs to the fibro-plastic, and is seated in the thickness of the dermis. In M. Ricord's Lessons on the Chancre is a detailed histological history of the nature of the induration, which I do not deem necessary to insert here, as there is nothing certain about it; and, furthermore, is not of the slightest practical importance. Those who wish to go deeper into the subject will find M. Robin's views in Ricord's Lessons on the Chancre. Suffice it to say, that M. Robin considers the induration to be principally located in the thickness of the dermis.

*Diagnosis.*—There are two modes to which French surgeons resort, both of which are almost absolutely impossible in America, where our population is extended over such a vast space; and again, this practice is impracticable with private patients. The means adopted by the French are inoculation and confronting the diseased and the person suspected. The latter course, as I said above, in the largest majority of cases, is impossible. For instance, a man contracting a chancre in New York or New Orleans, presents himself for treatment in St. Louis; it is easy to perceive at once that this, as a means of diagnosis, cannot be thought of. The former is practicable only in hospitals, as a private patient will not submit to such means of diagnosticating the affection, the more so as he can always find men who will take the responsibility of treating him on general principles, and satisfying himself with general results, frequently to the irreparable detriment of the unfortunate victim. Where, however, inoculation can be practiced, it is an invaluable assistant in making a diagnosis, as the exceptions to the rule, that the person who is affected cannot be reinoculated with the virus of the chancre, if it be an indurated one, are few; the cases in which they can be reinoculated are very rare. M. Clerc says he has inoculated two in one hundred. M. Fournier, M. Nadau des Islets, M. Laroyenne, M. Rollet and M. Roissau have all had almost similar results. When the chancre is accompanied by one of those cartilaginous-like indurations, it is not difficult to diagnosticate. Sometimes it is slow to make its appearance, obscure, and even in some cases it does not exist at all.

I here append MM. Bellehomme and Martin's differential of the characters of the chancre:

| INDURATED CHANCRE. | SOFT CHANCRE. |
|---|---|
| 1. There is an incubation, of which the average duration is fixed at 25 days. | 1. There is no period of incubation. |
| 2. It arises from the contagion of an indurated chancre, from suppurating, secreting, secondary lesions; sometimes from the blood of a patient affected with syphilis in the secondary stage. | 2. It arises from a simple chancre or a chancrous bubo. |
| 3. It is most frequently solitary. | 3. It is most frequently multiple. |
| 4. It is not inoculable to the person who has it, or to a person with constitutional syphilis. | 4. It is inoculable without end to the person who has it, or to any other person. The pus of the suppurating bubo is only inoculable when it is a chancrous one. |
| 5. It does not commence by a vesico-pustule, but by a simple excoriation, and in some cases by a papilla. | 5. It commences by a vesico-pustule. |
| 6. In the period called the *state*, the indurated is presented under the form of a superficial ulceration with inclined borders; this ulceration is covered, in part, by a false membrane; the borders are of a bright red; the form of the ulceration is generally regular; it suppurates very little. | 6. In the period called the *state*, the soft chancre is presented under the form of a tolerably profound ulceration, the bottom of which is filled with a kind of organic detritus mixed with pus. The borders are perpendicular, and separated on the edges from the subjacent tissues. |

| INDURATED CHANCRE. | SOFT CHANCRE. |
| --- | --- |
| 7. The indurated chancre is rarely painful. | 7. The soft chancre is almost always painful. |
| 8 The indurated chancre is accompanied 98 times in 100 with induration of its base, elastic and chancroid in its character, not having any of the inflammatory induration. | 8. The soft chancre is accompanied in some cases with an inflammatory induration, but never with the specific induration. |
| 9. The neighboring lymphatic ganglions of the indurated chancre become indurated, and give those polyganglionic adenophymata, which are chancroid and indolent, without any tendency to suppurate. | 9. The soft chancre is often accompanied by phlegmonous lymphatics. The bubo suppurates, and furnishes in some cases an inoculable pus. |
| 10. Not much local reaction; it has a tendency to cure itself; very rarely becomes phagedenic; it is regular in its course. | 10. The soft chancre is a tolerably grave local lesion. It has a great tendency to ulceration; very irregular in its course; does not tend to heal of itself like the indurated chancre. It is relatively very frequently complicated with phagedena and gangrenous state. |
| 11. The indurated chancre is the first apparent manifestation of the syphilitic diathesis; it is therefore the sign of general infection of the economy. Frequently before its cicatrization the first manifestations of the secondary stage come to light, such as roseola, etc. | 11. The soft chancre is purely a local accident. |
| 12. It is peculiar to the human family. | 12. It is transmissible to animals. |

A person can confound an indurated chancre in its birth with herpes, but the confusion can be avoided if they remember that herpes is always multiple, disposed in groups of vesicles to which succeed superficial excoriations, while the indurated chancre is almost always solitary. The herpes is a lesion at first vesiculous, then ulcerous, relatively dry, pseudo-membranous; besides, the herpes is never accompanied either by induration or sympathetic swelling of the glands. A mucous patch just commencing can also be mistaken for an indurated chanchre in debut; this can be avoided by examining the patient closely for some concomitant phenomena, such as roseola, sore throat, etc. Besides, the mucous patches are never indurated, and are very rarely accompanied with swelling of the glands, particularly if it is a relapse; again, the characteristic false membrane of the indurated chancre does not resemble in the least the gray pellicle which covers the mucous patch.

Small, gummy, isolated, ulcerating tumors sometimes resemble perfectly an indurated chancre in everything except the swelling of the glands. Indurated chancres have often been mistaken for epithelial cancers, and vice versa; differential diagnosis: the chancre is always accompanied with swelling of the glands, which takes place rarely with this form of cancer. The cancer is very slow in its course, and if the chancre exists beyond its ordinary duration it will be accompanied by secondary symptoms. Cicatrization of herpes, particularly with caustic potash, gives an indurated ulcer which often deceives the most expert, and makes them think that they have an indurated chancre to deal with. The soldiers often employ this means in order to obtain their discharge from the army; in any case the absence of the swelling of the glands will remove all doubt.

*Prognosis.*—As far as a local lesion the indurated chancre has not ordinarily any grave consequences, as has been said already in this letter. There are, however, several cases on record where patients have been rapidly debilitated, and finally hurried into the grave from this seemingly trivial but fearful scourge of man.

The chancre is followed more or less quickly by apparent manifestations of syphilitic diathesis, which constitutes what M. Ricord calls confirmed syphilis. It is very rare that six months pass without seeing these ordinary consequences of an indurated chancre.

The different secondary and tertiary symptoms only succeed each other at determined intervals. Mr. McCarthy, M. Bassereau, and M. Fournier, have all published statistical tables of these evolutions; they all present to each a remarkable concordance.

A TABLE, AFTER MM. BELLEHOMME AND MARTIN, GIVING THE TIME FOR THE APPEARANNCE OF SECONDARY AND TERTIARY MANIFESTATIONS.

| DENOMINATION OF THE ERUPTION. | The most ordinary time required for their appearance. | The earliest time of their appearance. | Latest time. |
|---|---|---|---|
| Roseola | 45 days. | 25 days. | 12 months. |
| Syphilide papulous | 65 days. | 28 days. | 12 months. |
| Mucous patches | 70 days. | 30 days. | 18 months. |
| Syphilide vesiculous | 90 days. | 55 days. | 6 months. |
| Syphilide pustulous | 80 days. | 45 days. | 4 years. |
| Rupia | 2 years. | 7 months. | 4 years. |
| Syphilitic iritis | 6 months. | 60 days. | 13 months. |
| Syphilitic sarcocele | 12 months. | 6 months. | 34 months. |
| Periostitis | 9 months. | 4 months. | 2 years. |
| Syphilide tuberculous | 3 to 5 years. | 3 years. | 20 years. |
| Syphilide of the serpiginous form | 3 to 5 years. | 3 years. | 20 years. |
| Gummy tumors | 4 to 6 years. | 4 years. | 15 years. |
| Affections of the nails | 4 to 6 years. | 3 years. | 22 years. |
| Free exostosis | 4 to 6 years. | 3 years. | 20 years. |
| Osteitis alteration of the bones and cartilages | 3 to 4 years. | 2 years. | 41 years. |
| Destruction of the bones of the palate | 3 to 4 years. | 2 years. | 20 years. |
| Secondary lesion of the thumb | 70 days. | 50 days. | 18 days. |

The several forms above of secondary and tertiary accidents succeed each other very near after the above order. Some writers go so far as to say that the above is nearly mathematical. "They follow," says Mr. McCarthy, "a gradual progression from the superficial layers to the most profound." It is useless to add that a mercurial treatment well administered can modify them much, retard, and prevent altogether their appearance.

M. Bellehomme thinks that there are benign and grave forms of syphilis. He says: "I believe that the volume of induration and its duration beyond the ordinary time are symptoms of a syphilis which will continue a long time, and the lesions will be numerous and persistent."

*Treatment.*—If we admit that the chancre is "the first manifestation of the syphilitic diathesis," it is absolutely useless to speak here of the former, but now defunct, abortive treatment of M. Ricord; if it is not hurtful, it is at least useless, as we have already seen that its tendency is to heal of its own accord. It should be kept clean in the commencement, and when it has reached the condition of "state," a light, exciting wash—aromatic wine—should be used, and it should be lightly touched with nitrate of silver. As for the general treatment I have but little to say; there are two medicines, mercury and iodide of potassium, used of course very extensively here. The first for the early stages, and the latter for other stages, tertiary.

A favorite prescription of corrosive sublimate, is as follows: Deutochloride of mercury, 1 gramme; pure water, 900 grammes; rectified alcohol, 100 grammes.

Here I must cease. If this compilation has benefitted any, I am fully paid for my time and trouble.

